# The Presence of *esat-6* and *cfp10* and Other Gene Orthologs of the RD 1 Region in Non-Tuberculous Mycobacteria, Mycolicibacteria, Mycobacteroides and Mycolicibacter as Possible Impediments for the Diagnosis of (Animal) Tuberculosis

**DOI:** 10.3390/microorganisms12061151

**Published:** 2024-06-05

**Authors:** Nomakorinte Gcebe, Tiny Motlatso Hlokwe, Agnes Bouw, Anita Michel, Victor P. M. G. Rutten

**Affiliations:** 1 Bacteriology Laboratory, Agricultural Research Council-Onderstepoort Veterinary Institute, Onderstepoort 0110, South Africa; hlokwet@arc.agric.za; 2 Division of Immunology, Department of Infectious Diseases and Immunology, Faculty of Veterinary Medicine, Utrecht University, 3584 CL Utrecht, The Netherlands; agnesbouw2020@gmail.com (A.B.); v.p.m.g.rutten@uu.nl (V.P.M.G.R.); 3 Department of Veterinary Tropical Diseases, Faculty of Veterinary Science, University of Pretoria, Onderstepoort 0110, South Africa; anita.michel@up.ac.za

**Keywords:** ESAT-6, Esx-1, CFP 10, RD1, *Mycobacteriaceae*, tuberculosis

## Abstract

The Esx-1 family proteins of the Type VII secretion systems of *Mycobacterium bovis* and *Mycobacterium tuberculosis* have been assessed and are frequently used as candidates for tuberculosis (TB) diagnosis in both humans and animals. The presence of ESAT-6 and CFP 10 proteins, which are the most immunogenic proteins of the Esx-1 system and have been widely investigated for the immunodiagnosis of tuberculosis, in some *Mycobacteriaceae* and in *Mycobacterium leprae*, poses limitations for their use in specific diagnoses of TB. As such, to improve the specificity of the ESAT-6/CFP 10-based cell-mediated immunity (CMI) assays, other proteins encoded by genes within and outside the RD 1 region of the esx-1 locus have been evaluated as candidate antigens for CMI, as well as to investigate humoral responses in combination with ESAT-6 and or CFP 10, with varying specificity and sensitivity results. Hence, in this study, we evaluated various non-tuberculous mycobacteria (NTM), *Mycolicibacterium*, *Mycolicibacter* and *Mycobacteroides* species genomes available on the NCBI database for the presence and composition of the RD1 region of the esx-1 locus. In addition, we also assayed by polymerase chain reaction (PCR) and sequencing of *Mycobacteriaceae* available in our culture collection for the presence and sequence diversity of *esxA* and *esxB* genes encoding ESAT-6 and CFP 10, respectively. Whole genome sequence (WGS) data analysis revealed the presence of RD 1 gene orthologs in 70 of the over 100 published genomes of pathogenic and non- pathogenic *Mycobcteriaceae* other than tuberculosis. Among species evaluated from our culture collection, in addition to earlier reports of the presence of *esxA* and *esxB* in certain *Mycolicibacterium*, *Mycolicibacterium septicum/peregrinum*, *Mycolicibacterium porcinum* and *Mycobacterium* sp. N845T were also found to harbour orthologs of both genes. Orthologs of *esxA* only were detected in *Mycobacterium brasiliensis*, *Mycolicibacterium elephantis* and *Mycolicibacterium flouroantheinivorans*, whereas in *Mycolicibacter engbackii*, *Mycolicibacterium mageritense* and *Mycobacterium paraffinicum*, only *esxB* orthologs were detected. A phylogenetic analysis based on *esxA* and *esxB* sequences separated slow-growing from rapidly growing bacteria. These findings strengthen previous suggestions that *esxA* and *esxB* may be encoded in the majority of *Mycobacteriaceae*. The role of the Esx-1 system in both pathogenic and non-pathogenic *Mycobacteriaceae* needs further investigation, as these species may pose limitations to immunological assays for TB.

## 1. Introduction

Tuberculosis, caused by members of the *Mycobacterium tuberculosis* complex (MTBC) bacilli, is an important zoonotic disease in both humans and animals. The disease has long been recognized worldwide as a significant health and economic risk. The main etiological agent in humans is *Mycobacterium tuberculosis*, and in animals TB is mainly caused by *Mycobacterium bovis* [1]. The *M. bovis* and *M. tuberculosis* Esx-1 encodes 23 genes, namely *esxA* to *esxW*, which occur in pairs, and the singleton *esxQ*, related to the WXG100 family of proteins, which is characterized by a size of ~100 amino acids and the presence of a Trp-Xaa-Gly (W-X-G) motif [2]. The role of the Esx-1 secretion system in virulence as well as immunogenicity has been well described [3]. The WXG100 family of proteins are among the most immunodominant antigens recognised by the animal and human immune system [4]. Region of difference (RD1) encodes Esx-1 locus proteins, of which ESAT-6 encoded by *esxA* and CFP 10 encoded by *esxB* are the most immunodominant proteins and most widely investigated for use in cell-mediated immunity (CMI)-based diagnoses of TB in animals and humans [5,6]. ESAT-6 and CFP 10 have been reported to lack sequence homology with other Esx family proteins of *M. bovis* and *M. tuberculosis*; they are absent in the *M. bovis* BCG vaccine strain and *M. microti*, which lack the RD1 region. Consequently, they have been evaluated for use as diagnostic markers to differentiate between TB infection and *M. bovis* BCG vaccination [5,6]. Now, according to the new classification, mycobacteria are reclassified into the following five clades: Tuberculosis-Simiae, consisting of members of the MTBC as well as pathogenic slow-growing (SG) NTM; Terrae clade, consisting of most of the non-pathogenic slow-growing species belonging to the *M. terrae* complex; the Triviale clade consisting of slow-growing *M. triviale* and *M. koreense*; the Chelonae-abscessuss clade, consisting of the pathogenic rapidly growing (RG) species; and lastly the Fortuitum–vaccae clade, consisting of most of the non-pathogenic rapidly growing species. The above were proposed to be the following genera: an amended *Mycobacterium*, *Mycolicibacter* gen. nov., *Mycolicibacillus* gen. nov., *Mycobacteroides* gen. nov and *Mycolicibacterium* gen. nov, respectively [7]. Orthologs of *esxA* and *esxB* are found in a number of species of the genera *Mycolicibacterium*, *Mycobacteroides*, *Mycobacterium* and *Mycolicibacter* [7,8], but so far investigations into the immunodominance of the homologs of ESAT-6 and CFP 10 and others have mainly focused on pathogenic non-tuberculous mycobacteria (NTM) that are phylogenetically related to the *Mycobacterium tuberculosis* complex (MTBC), such as *Mycobacterium kansasii* and *Mycobacterium marinum* as well as *Mycolicibacterium smegmatis* [6]. This is despite the reported occurrence of these genes in other species of the different *Mycobacteriaceae*, including non-pathogenic spp., among others in addition to *Mycolicibacterium smegmatis* [7,8], *Mycobacterium riyadhense* [8], *Mycobacterium gastri* [9], *Mycolicibacterium fortuitum*, *Mycolicibacterium malmesburyense*, *Mycolicibacterium komaniense*, *Mycolicibacterium.* spp. JLS and *Mycolicibacterium farcinogenes* [10]. Homologs of ESAT-6 (L-ESAT-6) and CFP 10 (L-CFP 10) in *M. leprae* have sequence similarities of 32% and 40%, respectively, to the *M. bovis* and *M. tuberculosis* proteins, and were reported to elicit adequate immune responses that may be used for the diagnosis of leprosy [11,12]. Despite these sequence differences, L-ESAT 6 and L-CFP 10 have also been shown to be recognised by T cells from TB patients [11,12]. Other proteins of the Esx-1 locus, including EspJ [13], and outside the Esx-1 locus, including Mb1992, Mb2031c, Mb2319, Mb2843, Mb2845c, Mb3212c, Rv0899 (OmpATB), EspC, EspF and others [13,14,15], either alone or in combination with ESAT 6/CFP 10, have also been investigated as candidate antigens for (animal) TB CMI assays with varying specificity and sensitivity results. Since (animal) TB diagnosis by CMI is complicated by the cross-reactivity of the immunodominant proteins with NTM orthologs, antibody-based assay development has therefore become a subject of research. Proteins such as MBP70 and MBP83, which are well-documented candidate antigens for humoral response; MBP63, which has been shown to induce both CMI and humoral response; and PE25 and PE41, which also form a dimer, and have been used as fusion antigens, have been evaluated as candidate antigens for recognition by Th2 lymphocytes in humoral immune response assays or in a combination of both CMI and humoral assays, including TB Enflerplex, Luminex technology and multi antigen print immune assay (MAPIA) [3]. Thus, there has been more of a focus on multiplex antigen assay development for antibody- and CMI-based diagnostics, as well as on the combination of both assays. A greater number of antigens may provide increased sensitivity, but specificity may be reduced due to cross reactions [3]. There has also been more of a focus on fusion of the ESAT 6/CFP 10 antigens with other antigens for improved sensitivity and specificity of CMI- and humoral-based assays, with these two antigens deemed important in (animal) TB diagnosis [16]

In the current study, we investigated the presence and arrangement of the RD1 region of Esx-1 in NTM, mycobacteroides, mycolicibacter and mycolicibacteria spp. This study is therefore believed to be able in the long run to assist in the development of TB diagnostic assays with improved specificity.

## 2. Materials and Methods

### 2.1. NCBI Database Search for RD1 Region Orthologs in Genomes of Mycobacteriaceae

*Mycolicibacterium smegmatis* (CP009494.1) sequences of *esxA* and *esxB* retrieved from the Smegmalist database (https://mycobrowser.epfl.ch/genes/; accessed on 20 January 2022), as well as *Mycolicibacterium fortuitum* (CP014258.1), *Mycobacterium leprae* (AL583917.1), *Mycobacterium kansasii* (CP006835.1), *Mycobacterium szulgai* (EU826486.1 and FJ014490.1, respectively), *Mycobacterium marinum* (CP024190.1), *esxA* and *esxB* sequences all retrieved from the NCBI database (https://www.ncbi.nlm.nih.gov/; accessed on 20 January 2022), were used as query reference sequences in an NCBI BLAST (blastn search). The maximum number of 100 hits for sequences producing significant alignments was selected in all the analyses. The BLAST results and the location/arrangement of the two genes, as well as translated gene orthologs of the RD1 region, were investigated using an NCBI nucleotide search (https://www.ncbi.nlm.nih.gov/nuccore/; accessed on 20 January 2022).

### 2.2. PCR Primer Design for esxA and esxB Genes’ Evaluation

To cover both slow growing (SG) as well as rapid growing (RG) *Mycobacteriaceae*, we used primer sequences designed from *M. bovis* and sequences derived from a rapidly growing, well-studied species, *M. smegmatis*, respectively. The first sets of primers were designed manually from the *M. smegmatis* MC^2^155 nucleotide sequences using NCBI primer BLAST. The *M. smegmatis* MC^2^155 sequences of *esxA* and *esxB* were derived from the *Smegmalist* database (https://mycobrowser.epfl.ch/genes/; accessed on 20 January 2022). The second sets of primers were designed manually from *M. bovis* AF2122/97 nucleotide sequences using NCBI primer BLAST. The *M. bovis* sequences of *esxA* and *esxB* were derived from Bovilist database (http://genolist.pasteur.fr/BoviList/genome.cgi?; accessed on 20 January 2022). The *M. bovis esxA* and *esxB* nucleotide sequences were also compared to *M. smegmatis* sequences by pairwise alignment using the Molecular Evolutionary Genetic Analysis (MEGA-X) platform [17]. The oligonucleotide sequences of the two genes are captured in Table 1. The *M. smegmatis*-derived primers were evaluated on *M. smegmatis* ATCC 14468 using PCR and sequencing, whereas *M. bovis*-derived primers were evaluated on *M. bovis* field isolates that were previously identified and characterised by Hlokwe et al. [1]. Each of the primer sequences was also evaluated for sequence match on NCBI BLAST, the using blastn algorithm.

### 2.3. Bacterial Species Subjected to Assessment for the Presence of esxA and esxB Using PCR-Sequencing

Forty-one isolates belonging to twenty-one bacterial species available in the NTM, mycolicibacteria and mycolicibacter culture collection of the ARC-OVI (Agricultural Research Council—Onderstepoort Veterinary Institute, Pretoria, South Africa) were included in this study. All isolates were derived from different sources, including soil, water, bovine nasal swabs and animal tissue, and had previously been identified to species level by Gcebe et al. [18]; Gcebe and Hlokwe [19]; and Gcebe et al. [20]. The *M. bovis* strains used for primer verification were previously identified by Hlokwe et al. [1]. In addition, American Type Culture Collection (ATCC) strains were used. Bacterial species used in this study, except for *M. bovis*, are presented in Table 2.

### 2.4. Polymerase Chain Reaction (PCR) for the Detection of esxA and esxB Gene Orthologs

Crude DNA was prepared from colonies and used as a template in polymerase chain reactions, as described by Gcebe et al. [18]. Briefly, individual colonies were suspended in PCR water and heated for 25 min at 100 °C. The supernatant was used as template DNA in the PCR protocols. The two primer pairs mentioned in Section 2.2, above, were used in the PCR assays (Table 1). The PCR conditions used for the amplification of the two gene fragments in separate PCR reactions are as follows: a 50 µL PCR reaction mixture was prepared, containing 28.5 µL de-ionised water, 3 µL MgCl_2_ (25 mM), 1 µL dNTP mix (10 mM), 4.75 µL of 10× PCR buffer (160 mM) (Tris -HCl, MgCl_2,_ Tween 20, (NH_4_)_2_, SO_4_), 0.75 µL Taq DNA Polymerase (5 U/µL) (Supertherm ^TM^), 1 µL of each forward and reverse primers (50 pmol) and 10 µL of DNA template. The PCR cycling parameters were as follows: 45 cycles of denaturation at 94 °C for 1 min, annealing at 60 °C for 1 min, and elongation at 72 °C for 1 min and final extension at 72 °C for 10 min. The PCR amplification products were electrophoresed on ethidium-stained 1.5% agarose gel and visualised under ultraviolet light (UV). The integrity of DNA of all the isolates that yielded negative results in all PCR assays for both *esxA* and *esxB* was tested by PCR targeting the *hsp65* gene, as described by Gcebe et al. [21].

### 2.5. Extraction and Purification of DNA from the Agarose Gel

For sequencing and to avoid the inclusion of non-specific PCR products if present, selected amplification products were excised as accurately as possible from the gel using a clean scalpel under transillumination (Spectroline UV Transulliminator, Model T312A). The weight of every excised DNA gel sample was recorded. DNA was extracted from the gel using the Qiagen gel extraction kit following the manufacturer’s Quick-Start protocol (QIAquick^®^ Gel Extraction Kit, Qiagen, Hilden, Germany). An aliquot of the extracted DNA was electrophoresed on agarose gel (1.5%) to confirm the success of DNA extraction.

### 2.6. Sequencing and Subsequent BLAST Analysis of the Orthologs of esxA and esxB Genes

Sequencing of the PCR products was performed at Inqaba Biotechnical Industries (Pty) Ltd. (Pretoria, South Africa) using an ABI sequencer. The PCR products were sequenced in both directions using the forward and reverse primer sequences that were initially used for amplification. Sequences from both strands were manually edited and pairwise alignments undertaken using the BioEdit Sequence alignment editor (version 7.1.9) and Molecular Evolutionary Genetics Analysis (MEGA X) platform [17]. The resulting consensus sequences were analysed on the NCBI platform for gene sequence identity/similarity using the Basic local alignment tool (BLAST) (www.blast.ncbi.nlm.nih.gov/Blast.cgi; accessed on 20 January 2022).

### 2.7. Phylogenetic Analysis

The phylogenetic analysis of 58 species of *Mycobacterium*, *Mycolicibacterium* and *Mycobacteriodes* combined was performed based on *esxA* and *esxB* gene sequences retrieved from the NCBI nucleotide database (https://www.ncbi.nlm.nih.gov/nucleotide; accessed on 20 January 2022). Multiple sequence alignments were performed using the MEGA-X platform [22]. Sequences were first trimmed manually at the 5′ and 3′ ends, so that they all began and ended at the same nucleotide position. Phylogenetic trees were created using the neighbour-joining method [23]. The neighbour-joining trees were verified using the maximum composite likelihood method, and one thousand bootstrap repeats were performed [24]. *M. bovis* AF2122/97 (LT 708304.1) and *Mycobacterium shinjukeunse* JCM14233 (AP.022575.1) were used as outgroup sequences.

## 3. Results

### 3.1. Evaluation of the Designed Primers for PCR

**Figure 1:**
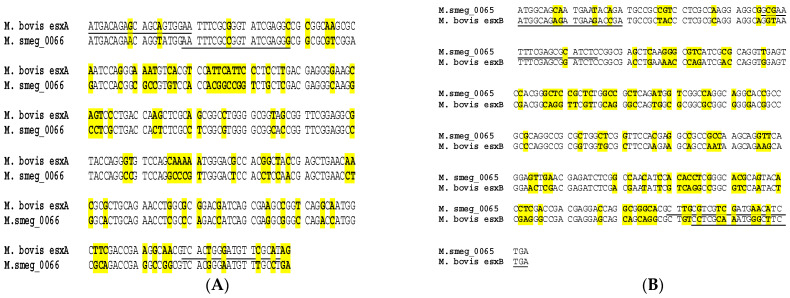
(**A**) Alignment of *M. bovis esxA* and *M. smegmatis msmeg_0066* gene sequences encoding for Esat-6. (**B**) Alignment of *M. bovis esxB* and *M. smegmatis msmeg_0065* gene sequences encoding for CFP 10. Sequence differences are highlighted in yellow, and positions of oligonucleotides are underlined.

The amplification of the *esxA* and *esxB* gene fragments was shown in the *M. smegmatis* ATCC 14468 strain, resulting in products of approximately 250 bp and 270 bp for *esxB* and *esxA*, respectively, as well as in *M. bovis* field isolates, resulting in products of approximately 290 bp and 300 bp for *esxA* and *esxB*, respectively. Sequence data BLAST search results for the respective gene fragment sequences indicated that the amplified sequence using *esxA* primers was 100% identical to the *M. smegmatis* INHR2 strain (CP009496.1), position 87348–87617 in the genome. The amplified sequence when *esxB* primers were used was also identical to the *M. smegmatis* INHR2 strain (CP009496.1), at position 87041–87295 in the genome. Likewise, the verification of *M. bovis-*derived primers on *M. bovis* field isolates from our culture collection [1] revealed 100% sequence identity to *esxA* and *esxB* of the *M. bovis* AF2122/97 genome, at position 4288929–4289216 and position 4288594–42888896, respectively. The alignment of the *M. bovis* AF2122/97 *esxA* and *esxB* and *M. smegmatis* MC^2^155 *esxA* ortholog (Msmeg 0066) and *esxB* ortholog (Msmeg 0065) revealed 28% and 33% sequence divergence (Figure 1A,B). The combined results of the NCBI BLAST using *M. smegmatis-*derived *esxA* forward and reverse primers revealed sequence identities to *esxA/WXG100* family gene fragments of the following species, i.e., different strains of *M. smegmatis*, *Mycolicibacterium hassiacum*, *Mycolicibacterium goodii*, *Mycolicibacterium thermoresistible*, *Mycolicibacterium septicum*, *Mycolicibacterium boeneckei*, *Mycolicibacterium farcinogenes*, *Mycolicibacterium* sp. VKM Ac-1817D, *Mycolicibacterium fortuitum* and *Mycolicibacterium senegalense.* Similarly, the NCBI BLAST of *M. smegmatis-*derived *esxB* primers revealed sequence identities to different strains of *M. smegmatis*, *M. goodii*, *M. boenickei*, *M. farcinogenes*, *Mycolicibacterium lentiflavum*, *Mycolicibacterium malmoense*, *Mycolicibacterium parakoreensis* and *Mycobacterium* subspecies *paratuberculosis esxB/WXG 100 family* gene fragments. On the other hand, the NCBI BLAST of the *M. bovis-*derived *esxA* primers revealed matches to *M. tuberculosis* complex (MTBC) species, *M. kansasii* and *Mycobacterium ostraviense esxA* fragments. The *M. bovis-*derived *esxB* primer NCBI BLAST showed sequence matches to those in the MTBC species, as well as in *Mycolicibacterium novocastrense*, *Mycobacterium pseudoshotsii*, *Mycobacterium shotsii* and *M. kansasii.*

### 3.2. The Presence of esxA and esxB in NTM, Mycolicibacteria, Mycolicibacter and Mycobacteroides Isolates as Determined by PCR and Sequencing

Among the reference strains shown in Table 3, both *esxA* and *esxB* genes were amplified from the DNA of *M. fortuitum* (ATCC 6481) and *M. smegmatis* (ATCC 14468) using *M. smegmatis-*derived primers. In *M. moriokaense* (ATCC 43059), neither of the genes were detected using either *M. smegmatis-* or *M. bovis-*derived primers for amplification. Field isolates belonging to *M. fortuitum*, *M. mageritense*, *Mycolicibacterium* sp. N845T, the *Mycolicibacterium septicum/M. peregrinum* group, *Mycolicibacterium paraffinicum* and *Mycolicibacterium porcinum* were also found to harbour both *esxA* and *esxB* genes. Only the *esxA* gene was detected in DNA of isolates belonging to *Mycolicibacterium flouroanthenivorans*, *Mycolicibacterium elephantis* and *Mycolicibacterium brasiliensis* using *M. smegmatis-*derived primers. Only the *esxB* gene was identified in DNA of *Mycolicibacter engbaeckii* using *M. bovis-*derived primers. The amplification of neither *esxA* nor *esxB* genes was observed in isolates belonging to *Mycolicibacterium acapulcensis*, *Mycolicibacterium chitae*, *Mycolicibacterium confluentis*, *Mycolicibacterium vaccae/M. vanbaalenii*, *Mycolicibacterium parafortuitum*, *Mycolicibacterium austroafricanum*, *Mycolicibacterium madagascariense*, *Mycolicibacterium komaniense*, *Mycolicibacterium malmesburyense*, *Mycolicibacterium neoaurum* and *Mycolicibacterium moriokaense.* Figures S1 and S2 show examples of amplified *esxA* and *esxB* gene fragments, respectively, using *M. smegmatis*-derived primers, while Figures S3 and S4 are examples of gel electrophoresis images for amplified *esxA* and *esxB*, respectively, using *M. bovis*-derived primers.

### 3.3. The Presence of RD1 Region Orthologs in NTM, Mycolicibacteria and Mycobacteroides Genomes: An NCBI Database Search

Using NCBI BLAST searches employing *Mycolicibacterium smegmatis*, *Mycolicibacterium fortuitum*, *Mycobacterium leprae, Mycobacterium kansasii*, *Mycobacterium szulgai* and *Mycobacterium marinum esxA* and *esxB* as reference query sequences, we observed that these two genes and orthologs were harboured in the genomes of at least 70 *Mycobacteriaceae*, including NTM (*n* = 27), *Mycolicibacterium* (*n* = 42) and *Mycobacteroides* (*n* = 1) species, as shown in Table 4. The two genes were also detected as individual gene coding sequences in *M. szulgai*, as no data for whole genome sequences for this species were available. The NCBI nucleotide data search revealed the occurrence of the RD1 region in all the 70 *Mycobactericeae* species except for *Mycolicibacterium branderi*, where only *esxA* was found and no ortholog of *esxB*. Predicted protein products of the genes within the RD1 region for different species are captured in Table 4. The WGS of *Mycobacterium riyadhense* was only available on the NCBI nucleotide database as a summary, and thus the RD1 region analysis could not be carried out for this bacillus.

### 3.4. The esxA- and esxB-Based Phylogenetic Analysis

**Figure 2:**
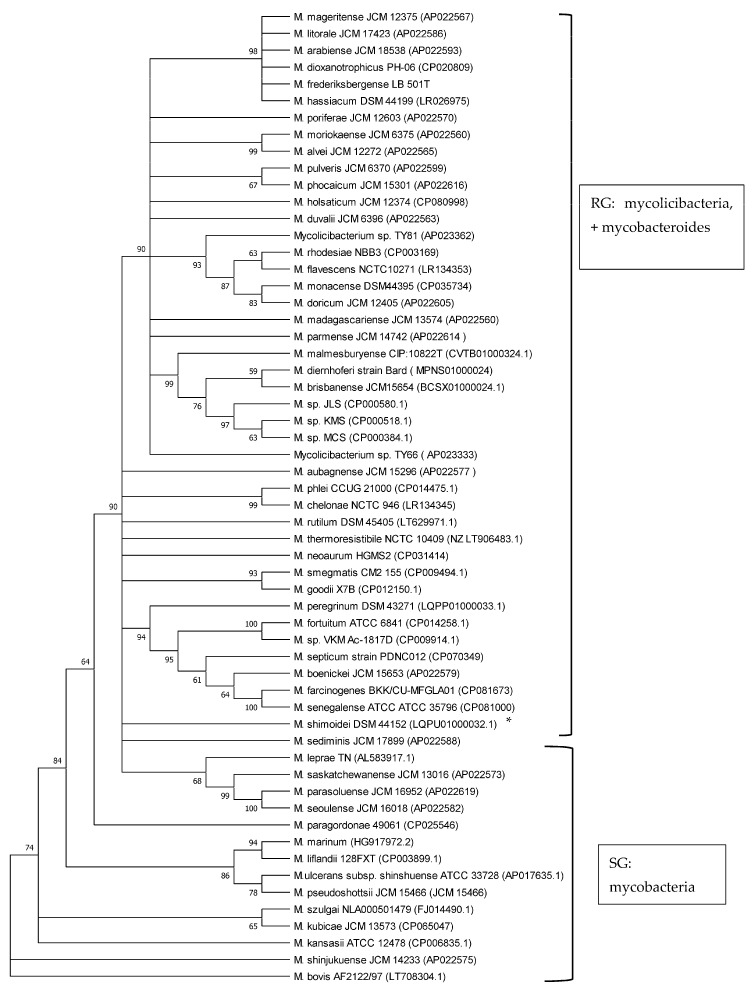
Phylogenetic tree illustrating the evolutionary relationships of *Mycobacteriaceae* based on *esxA* gene sequences constructed using the neighbour-joining method. All the sequences were retrieved from Genbank. Branches corresponding to partitions reproduced in less than 50% bootstrap replicates are collapsed. The percentage of replicate trees in which the associated taxa clustered together in the bootstrap test (1000 replicates) are shown next to the branches. * is SG bacterium. All RG are *Mycolicibacterium species* and *Mycobacteroides chelonae;* all SG are *Mycobacterium* species.

**Figure 3:**
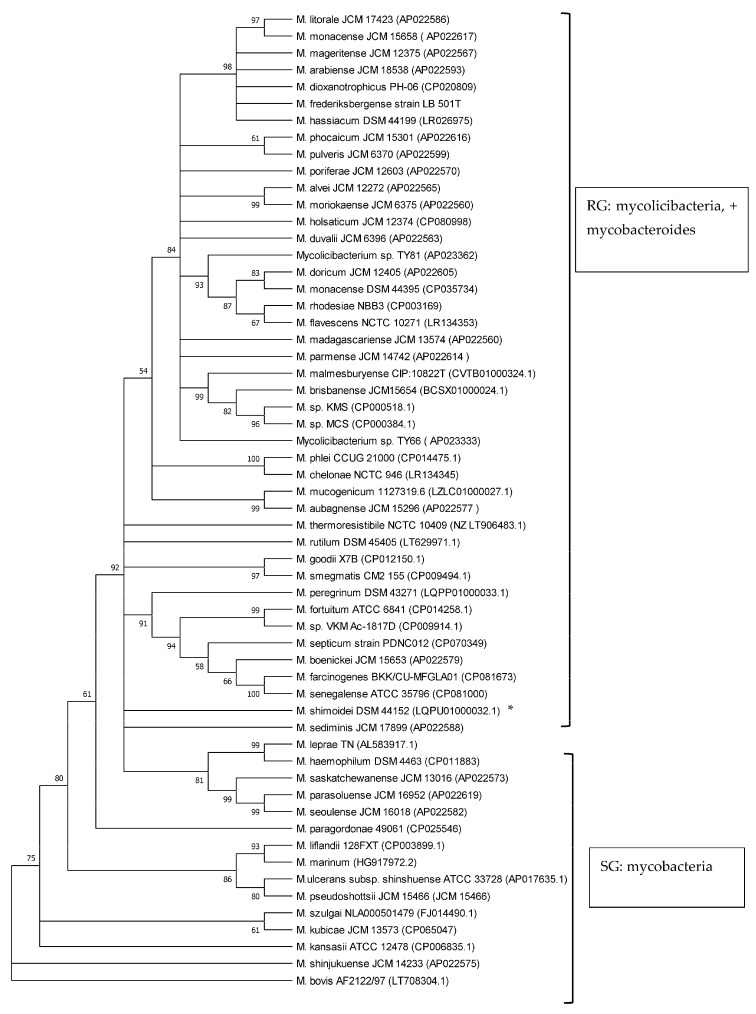
Phylogenetic tree illustrating the evolutionary relationship of *Mycobacteriaceae* based on *esxB* gene sequences constructed using the neighbour-joining method. All the sequences were retrieved from Genbank. Branches corresponding to partitions reproduced in less than 50% bootstrap replicates are collapsed. The percentage of replicate trees in which the associated taxa clustered together in the bootstrap test (1000 replicates) are shown next to the branches. * is SG bacterium. All RG are *Mycolicibacterium species* and *Mycobacteroides chelonae;* all SG are *Mycobacterium* species.

Phylogenetic analyses of mycobacteria, mycolicibacteria and mycobacteroides, based on *esxA* and *esxB* sequences, respectively, revealed a clear separation of slow-growing *Mycobacterium* species and rapidly growing *Mycolicibacterium* and *Mycobacteroides* species, supported by up to 100% bootstrap values (Figure 2 and Figure 3). *M. chelonae*, the only species of the *Mycobacteroides* genus included in the analysis, clustered with *Mycolicibacterium* spp. and was found to be closer to *Mycolicibacterium phlei*. This clustering was supported by 99% and 100% bootstraps in *esxA* and *esxB* sequence-based trees, respectively. Sequences that were too divergent (≤50% to any of the included sequences) were excluded from this analysis.

## 4. Discussion

The presence of *esxA* and *esxB* genes and their protein products in NTM has been a subject of research, following the detection of these two genes and immunogenic epitopes of their protein products in some pathogenic NTMs closely related to MTBC, such as *M. kansasii* and *M. marinum*. This has been a great concern, as these protein products have been broadly used as markers in the cell-mediated immunodiagnosis of both human and animal TB, since they were thought to be specific to members of the MTBC [6,10,25]. As such, other proteins have been investigated for use as markers for the detection of CMI and humoral responses or as a combination of both for the diagnosis of (animal) TB [3,16].

To further understand the role of NTM, *Mycobacteroides*, *Mycolicibacterium* and *Mycolicibacter* spp. in host immune responsiveness, we set up and screened isolates available in our biobank for *esxA* and *esxB* using PCR and sequence analysis of these two genes. We also analysed genomes in the NCBI database for the presence of the RD1 region orthologs of the esx-1 locus using *esxA* and *esxB* as query sequences in the NCBI BLAST analysis. Previous studies that reported NTM orthologs of *esxA* and *esxB* used PCR with primers designed from *M. tuberculosis*/*M. bovis* to screen SG as well as RG species [8,24,25,26]. Disparities in findings from some of these studies were seen as some reported the presence of these genes and others the absence in similar species, probably due to differences in primers used for amplification or PCR failures [8,24,25,26]. We therefore used primer sequences derived from a rapidly growing species, *M. smegmatis*, as well as primers designed from *M. bovis* for the amplification of the two genes for improved sensitivity. Employing the PCR-sequencing approach using primers derived from *M. smegmatis*, we confirmed the occurrence of *esxA* and *esxB* in *M. smegmatis* and *M. fortuitum* reference ATCC strains as well as field isolates, as reported previously [8,24]. We also showed the presence of the two genes in field isolates belonging to rapidly growing (RG) species, including, *Mycobacterium* sp. N845T, the *M. septicum/M. peregrinum* group and *M. porcinum*. It should be noted that *Mycobacterium* sp. N845T is not a validly published species; however, it is phylogenetically closer to RG species, as previously determined by 16S rRNA gene analysis [20]. Therefore, the amplification of both the *esxA* and *esxB* genes confirmed that some non-pathogenic RG species also harbour *esxA* and *esxB* gene orthologs.

Only *esxB* orthologs were detected in *M. paraffinicum*, *M. engbaeckii* and *M. mageritense* and only *esxA* orthologs were detected in *Mycolicibacterium elephantis*, *M. brasiliensis* and *Mycolicibacterium flouroantheinivorans*. To obtain a better understanding of RD1 region orthologs in *Mycobacteriaceae* other than tuberculosis, we analysed whole genome sequences of NTM, *mycolicibacteria*, *mycolicibacter* and *mycobacteroides* available in the public databases for the presence of genes of the RD1 region. Currently, there are more than 230 NTM, *Mycolicibacterium* and *Mycobacteroides* species combined on the List of Prokaryotic names with Standing in Nomenclature (LPSN) [27], and more than 100 genomes of these genera are available in the NCBI genome (http://ncbi.nhlm.gov/genome; accessed on 20 January 2022) and the NCBI Bio-project (http://ncbi.nhlm.gov/bio-project; accessed on 20 January 2022) databases. In this study, the RD 1 region orthologs were found to be harboured in genomes of more than 70 species. These include genomes of slow-growing (SG) pathogenic NTM, which are known to harbour RD1 region orthologs, among others *M. marinum* and *M. kansasii*; and genomes of non-pathogenic, at most opportunistic pathogenic RG spp., including, among others, *M. fortuitum*, *M. smegmatis*, *Mycolicibacterium* spp. JLS and *Mycolicibacterium* spp. MCS, as well as genomes of species not previously reported to harbour the RD 1 region [6,7,10,20]. *Mycolicibacterium pulveris*, *Mycobacteroides chelonae*, *M. moriokaense*, *Mycolicibacterium. boenickei*, *Mycobacterium seoulense* and *Mycobacterium paraseoulense* are among those genomes of species not previously reported to harbour the RD 1 region, but in this current study, RD 1 orthologs are reported. All these genomes were sequenced in other studies and submitted to the NCBI databases between the years 2013 and 2021 (http://ncbi.nhlm.gov/genome; http://ncbi.nhlm.gov/bio-project; accessed on 20 January 2022) (Table 4). The approach employed in the current study, using NCBI BLAST to investigate the presence of the RD 1 region, may not have been exhaustive of all NTM, *mycolicibacteria* and *mycobactoides* that may harbour these genes, due to the possible large sequence diversity of *esxA* and *esxB*, which were used as reference markers for the investigations.

The identification of the RD 1 region of the Esx-1 locus in more than 70 sequenced genomes of *Mycobacteriaceae*, including NTM, *mycolicibacteria* and *mycobacteroides*, confirms that this locus may be typical of the *Mycobacteriaceae* family [8,10]. The location of *esxA* and *esxB* genes next to each other in the genomes of the analysed species, as well as the presence of neighbouring gene orthologs of the RD1 region, including the recently investigated *espJ* as an additional marker for the immunodiagnosis of (animal) TB, calls for further investigation regarding their expression and the secretion of their protein products. Should these immunogenic protein homologs be expressed, secreted and recognised by T-cells, they may impact on the specificity of the diagnosis of (animal) TB by CMI assays that use either purified protein derivatives (PPD) or specific antigens such as ESAT-6, CFP10 and other antigens encoded in the RD 1 region, as previously experienced [28,29].

The use of 16S rRNA gene sequence analysis, as well as the analysis of other *Mycobacterium* housekeeping genes like *rpoB*, *hsp65*, *ITS* and *sodA*, has long been proven to be a robust tool to study phylogenetic relationships and the classification of the now-amended genus *Mycobacterium*, *Mycolicibacterium* and *Mycolicibacter*, *Mycobacteroides* and *Mycolicibacillus* spp. by growth rate, as well as to investigate their pathogenic potential [7,20,30,31,32,33]. Previous studies have shown that *esxA* and *esxB* sequences have the potential to be employed in phylogenetic analyses of the genera and their classification according to growth rate, i.e., as SG and RG species [8,20]. In the current study, the phylogenetic analysis of 58 *Mycobacteriaceae* including *Mycobacterium*, *Mycobacteroides* and *Mycolicibacterium* using *esxA* and *esxB* sequences grouped these into slow-growing and rapidly growing organisms. These findings are in line with results from other studies employing markers such as 16S rRNA, *hsp65*, *ITS* and *sodA* that demonstrated a robust phylogenetic classification *of Mycobacteriaceae* by growth rate [20,30,31,32,33]. Recently, core genomes were employed for the phylogenetic classification and reclassification of mycobacteria into five new genera [7]. Our analysis separated mycolicibacteria from mycobacteria. However, in contrast to the classification using core genomes, as described by Gupta et al. [7], in our study, using *esxA* and *esxB* as phylogenetic markers, *Mycobacteroides chelonae* could not be separated from the *Mycolicibacterium* clade and was closer to *Mycolicibacterium phlei* in both trees. The separation of RG from SG using *esxA* and *esxB* further strengthens earlier suggestions about the use of these two genes as potential phylogenetic markers for mycobacteria and now-amended mycobacteria, mycolicibacteria, mycobacteroides, mycolicibacilli and mycolicibacter [8,20].

In conclusion, we have demonstrated the presence of *esxA* and *esxB* orthologs as well as the presence of gene orthologs of the RD1 region in 70 *Mycobacteriaceae* including NTM, *Mycolicibacterium* and *Mycobacteroides* species’ full genomes using an in silico genome search approach. We also demonstrated the presence of these genes in species of the three genera and in *Mycolicibacter engbaekii* using PCR and sequencing techniques. In addition, whole genome sequence analysis provided a better understanding of the presence of *esxA* and *esxB* as well as the RD1 orthologs in *Mycobacteriaceae* other than tuberculosis compared to the PCR amplification. The characterization of *Mycobacteriaceae* for the presence as well as sequences of genes such as *esxA* and *esxB* and other genes of the esx-1 locus that play an important role in immune responses should form part of future studies describing new species. This warrants further investigations of the actual expression and secretion of these antigen homologs, as well as their role in cross-reactive immune responses. Lastly, *esxA* and *esxB* may be useful tools for the phylogenetic classification of species of *Mycobacteriaceae*.

## Figures and Tables

**Table 1:** Primer sequences designed and used for the amplification of *esxA* and *esxB*.

Gene (Mycobacteriaceae)	Primer Sequences	Position in the Gene	Expected Product Size in (bp)
*esxA/Msmeg_0066* (*M. smegmatis-*)	Forward: 5′ aatttcgccggtatcgaggg 3′ Reverse: 5′ caggcaaacattcccgtgac 3′	19–38 287–268	269 bp
*esxB/Msmeg_0065* (*M. smegmatis*)	Forward: 5′ gcgaatttcgagcgcatctc 3′ Reverse: 5′ gatgttcatcgacgacgcaag 3′	46–65 300–280	255 bp
*esxA* (*M. bovis*)	Forward: 5′ atgacagagcagcagtggaa 3′ Reverse: 5′ ctatgcgaacatcccagtga 3′	1–20 288–268	288 bp
*esxB* (*M. bovis*)	Forward: 5′ atggcagagatgaagacaga 3′ Reverse: 5′ tcagaagcccatttgcgagg 3′	1–20 303–283	303 bp

**Table 2:** The origin of *Mycobacteriaceae* used in the study.

Isolate Origin	Mycobacteriaceae	References
Reference strain	*Mycolicibacterium smegmatis*	ATCC 14468
Reference strain	*Mycolicibacterium fortuitum*	ATCC 6481
Reference strain	*Mycolicibacterium moriokaense*	ATCC 43059
Bovine nasal swab; soil	*Mycolicibacterium moriokaense* field isolate (*n* = 2)	[19]
Bovine organ	*Mycolicibacterium mageritense* (*n* = 1)	[19]
Guppy fish, soil	*Mycolicibacterium fortuitum* (*n* = 2)	[17,19]
Water	*Mycolicibacterium austroafricanum* (*n* = 1)	[19]
Soil, koi fish and natal ghost frog	*Mycolicibacterium septicum/peregrinum complex* (*n* = 3)	[17,19]
Bovine swab	*Mycolicibacterium komaniense* (*n* = 1)	[11]
Bovine swab	*Mycolicibacterium. malmesburyense* (*n* = 1).	[11]
Bovine nasal swab	*Mycolicibacterium vaccae/Mycolicibacterium. vanbaalenii* (*n* = 5)	[19]
Bovine swab	*Mycolicibacterium madagascariense* (*n* = 2)	[19]
Bovine nasal swab, soil, lion tissue	*Mycolicibacterium acapulcensis* (*n* = 3)	[19,20]
Bovine nasal swab; lion tissue	*Mycolicibacterium. elephantis* (*n* = 2)	[19,20]
Bovine nasal swab	*Mycobacteroides chitae* (*n* = 1)	[19]
soil	*Mycolicibacterium confluentis* (*n* = 1)	[19]
Water, soil	*Mycobacterium paraffinicum* (*n* = 2)	[19]
Soil, bovine nasal swab,	*Mycolicibacterium neoaurum* (*n* = 3)	[19]
Soil, water	*Mycolicibacter engbaeckii* (*n* = 3)	[19]
Bovine nasal swab, soil bush buck tissue	*Mycolicibacterium parafortuitum* (*n* = 3)	[19]
Lion tissue	*Mycolicibacterium flouroanthenivorans* (*n* = 1)	[20]
Sea horse	*Mycobacterium sp. N845T* (*n* = 1)	[17]
Natal ghost frog	*Mycolicibacterium porcinum* (*n* = 1)	[19]
Blesbok tissue	*Mycobacterium brasiliensis* (*n* = 1)	[20]

**Table 3:** Assessment of Mycobacteriaceae for the presence of *esxA* and *esxB* by PCR.

Isolate Origin	Mycobacteriaceae	PCR Results			
		*esxA* (*M. smegmatis* Primers)	*esxB* (*M. smegmatis* Primers)	*esxA* (*M. bovis* Primers)	*esxB* (*M. bovis* Primers)
Reference strain	*Mycolicibacterium smegmatis ATCC* 14468	+	+	ND	ND
Reference strain	*Mycolicibacterium fortuitum* ATCC 6481	+	+	ND	ND
Reference strain	*Mycolicibacterium moriokaense* ATCC 43059	-	-	-	-
Bovine nasal swab; soil	*Mycolicibacterium moriokaense* field isolate (*n* = 2)	-	-	-	-
Bovine organ	*Mycolicibacterium mageritense* (*n* = 1)	-	+	ND	ND
Guppy fish, soil	*Mycolicibacterium fortuitum* (*n* = 2)	+	+	ND	ND
Water	*Mycolicibacterium austroafricanum* (*n* = 1)	-	-	-	-
soil, koi fish and natal ghost frog	*Mycolicibacterium septicum/peregrinum complex* (*n* = 3)	+	+	ND	ND
Bovine swab	*Mycolicibacterium komaniense* (*n* = 1)	-	-	-	-
Bovine swab	*Mycolicibacterium. malmesburyense* (*n* = 1).	-	-	-	-
Bovine nasal swab	*Mycolicibacterium vaccae/Mycolicibacterium. vanbaalenii* (*n* = 5)	-	-	-	-
Bovine swab	*Mycolicibacterium madagascariense* (*n* = 2)	-	-	-	-
Bovine nasal swab, soil, lion tissue	*Mycolicibacterium acapulcensis* (*n* = 3)	-	-	-	-
Bovine nasal swab; lion tissue	*Mycolicibacterium. elephantis* (*n* = 2)	+	-	-	-
Bovine nasal swab	*Mycobacteroides chitae* (*n* = 1)	-	-	-	-
Soil	*Mycolicibacterium confluentis* (*n* = 1)	-	-	-	-
Water, soil	*Mycobacterium paraffinicum* (*n* = 2)	-	+	-	
Soil, bovine nasal swab,	*Mycolicibacterium neoaurum* (*n* = 3)	-	-	-	-
Soil, water	*Mycolicibacter engbaeckii* (*n* = 3)	-	-	-	+
Bovine nasal swab, soil Bush buck tissue	*Mycolicibacterium parafortuitum* (*n* = 3)	-	-	-	-
Lion tissue	*Mycolicibacterium flouroanthenivorans* (*n* = 1)	+	-	-	-
Sea horse	*Mycobacterium* sp. *N845T* (*n* = 1)	+	+	ND	ND
Natal ghost frog	*Mycolicibacterium porcinum* (*n* = 1)	+	+	ND	ND
Blesbok tissue	*Mycobacterium brasiliensis* (*n* = 1)	+	-	-	-

+: Amplification of the expected size; -: no amplification observed; ND: not done.

**Table 4:** Region in NTM, mycolicibacteria and mycobacteroides.

Mycobacteriaceae	RD1 Translated Gene Orthologs	*esxA* Ortholog Genome Co-Ordinates	*esxB* Ortholog Genome Co-Ordinates
*M. tuberculosis* H37Rv (Reference strain)	EccCb (Rv3871), PE35, PPE68, Cfp 10, Esat 6, EspI, EccD1, EspJ, EspK (Rv3879/)	4352609…4352896	4352274…4352576
*M.bovis* AF2122/97 (Reference strain)	EccCb (Mb3901), PE35, PPE68, Cfp 10, Esat 6, EspI, EccD1, EspJ, EspK (Mb3909	4288929…4289216	4288594…4288896
*Mycolicibacterium smegmatis* INHR2 (CP009496)	EccC_b, PE family, PPE family, Cfp 10, Esat 6, ParA, eccD, hypothetical protein, hypothetical protein	87330…87617	86996…87298
*Mycolicibacterium goodii* strain X7B (CP012150)	EccC_b, PE family, PPE family, EsxB, EsxA, ParA, EccD, hypothetical protein, hypothetical protein	1511158…1511445	1510825…1511127
*Mycolicibacterium fortuitum* CT6 (CP011269)	FtsK/SpoIIIE, PE family, PPE family, EsxB, EsxA, FlhG (RD 1 region associated), EccD, hypothetical protein, WXG100 family	57283…57570	56950…57252
*Mycolicibacterium* sp. VKM Ac-1817B (CP009914)	EccC_b, PE35, PPE68, esxB, esxA, EspI, EccD, hypothetical protein, WXG100 family	55533…55820	55200…55502
*Mycolicibacterium alvei* JCM 12272 (AP022565)	WXG100 family, hypothetical protein, EccD, ParA, EsxA, esxB, PPE family, PE35, EccC_b1	3587250…3587537	3587568…3587870
*Mycolicibacterium senegalense* ATCC 35796 (CP081000)	EccC_b, PE, PPE, EsxB, EsxA, ATPase, EccD, DUF 433 domain containing protein, hypothetical protein	98199…98486	97866…98168
*Mycolicibacterium farcinogenes* strain BKK/CU-MFGLA001 (CP081673)	Hypothetical protein, DUF4333 containing domain, EccD, ATPase, EsxA, EsxB, PPE family, PE family, EccC_b	197853…198140	198171…198473
*Mycolicibacterium boenickei* JCM 15653 (AP022579)	EccC_b1, PE35, PPE family, EsxB, EsxA, ParA, EccD, hypothetical, espA/espE family	1359841…1360128	1359507…1359809
*Mycolicibacterium dioxanotrophicus* strain PH06 (CP020809)	EccC_b, PE family, PPE family, EsxB, esxA, ParA, EccD, EspA/EspE, hypothetical	7455171…7455458	7455491....7455790
*Mycolicibacterium monacense* DSM 44395 (CP035734)	EccC_b, PE family, PPE family, EsxB, EsxA, ParA, EccD, hypothetical protein, YbaB/EbfC family	96584…96871	96254…96553
*Mycolicibacterium* sp. KMS (CP000518)	FtsK/SpoIIIE, PE family, PPE family, EsxB, EsxA, hypothetical protein, hypothetical protein of unknown function DUF571, hypothetical protein, hypothetical protein	91103…91390	90773…91072
*Mycolicibacterium* sp. MCS (CP000384)	FtsK/SpoIIIE, PE family, PPE family, EsxB, EsxA, hypothetical protein, hypothetical protein of unknown function DUF571, hypothetical protein, hypothetical protein	83763…84050	83433…83732
*Mycolicibacterium thermoresistible* NCTC 10409 (LT906483)	EccC_b, PE family, PPE family, EsxB, EsxA, ATPase, EccD, Protein of uncharacterised function (DUF2580), LppJ	71381…71668	71048…71350
*Mycolicibacterium* sp. JLS (CP000580)	FtsK/SpoIIIE, PE family, PPE family, EsxB, EsxA, hypothetical protein, hypothetical protein of unknown function DUF571, hypothetical protein, hypothetical protein	67008…67295	66678…66977
*Mycolicibacterium litorale* JCM 17423 (AP022586)	DNA binding protein (YbaB/EbfC family), hypothetical protein, ParA, EsxA, EsxB, PPE family, PE35, EccC_b1	4319168…4319455	4319486…4319785
*Mycolicibacterium doricum* JCM 12405 (AP022605)	EccC_b1, PE35, PPE family, EsxB, EsxA, ParA, EccD, tRNA-Cys, DNA binding protein	3955449…3955736	3955107…3955418
*Mycolicibacterium mageritense* JCM 12375 (AP022567)	WXG100, hypothetical protein, EccD, ParA, esxA, esxB, PPE family, PE35, EccC_b1	2348174…2348461	2348498…2348791
*Mycolicibacterium hassiacum* DSM 44199 (LR026975)	Hypothetical protein, WXG100, EccD1, EspI, EsxA, esxB, PPE family, PE35, EccC_b	4437958…4438245	4438279…4438581
*Mycolicibacterium neoaurum* strain MN2019 (CP074376)	Hypothetical protein, DNA binding protein (YbaB/EbfC family), EccD, ParA, EsxA, EsxB, PPE family, PE35, EccC_b	671381…671661	671698…672000
*Mycolicibacterium holsaticum* JCM 12374 (CP080998)	Hypothetical protein, acyl-CoA dehydrogenase family, EccD, EsxA, EsxB, PPE, PE, EccC_b	5568275…5568562	5568599…5568901
*Mycolicibacterium sediminis* JCM 17899 (AP022588)	EccC_b1, PE35, PPE family, EsxB, EsxA, ParA, EccD, hypothetical protein, hypothetical protein	1372541…1372828	1372201…1372503
*Mycolicibacterium rutilum* strain DSM 45405 (LT629971)	EccC_b, PE family, PPE family, EsxB, EsxA, ATPase, EccD, Uncharacterised DUF427 protein, Acyl-CoA dehydrogenase	1485513…1485800	1485171…1485479
*Mycolicibacterium pulveris* JCM 6370 (AP022599)	Hypothetical protein, Acyl-CoA dehydrogenase, EccD, ParA, esxA, esxB, PPE, PE35, EccC_b1	2281561…2281848	2281891…2282193
*Mycolicibacterium diernhoferi* ATCC 19340 (CP080332)	EccC_b, PE family, PPE family, EsxB, EsxA, ATPasem EccD, YbaB/EbfC family, hypothetical protein	235851…236138	235521…235820
*Mycolicibacterium madagascariense* JCM 13574 (AP022610)	Hypothetical protein, WXG100, EccD, ATPase, EsxA, EsxB, PPE family, PE35, EccC_b1	5609593…5609880	5609899…5610201
*Mycolicibacterium moriokaense* JCM 6375 (AP022560)	Hypothetical protein, hypothetical protein, EccD, ParA, EsxA, EsxB, PPE family, PE family, EccC_b1	411553…411837	411869…412171
*Mycolicibacterium arabiense* JCM 18538 (AP022593)	Efflux pump, IF-2, EccD, ParA, esxA, esxB, PPE family, PE family, EccC_b1	3481600…3481893	3481924…3482226
*Mycobacterium grossiae* strain DSM 104744 (CP043474)	DNA binding protein, hypothetical protein, RpfE, EccD, ATPase, EsxA, EsxB, PPE family, PE family, EccC_b	1279007…1279294	1279330…1279632
*Mycolicibacterium fluoranthenivorans* strain 2A (CP059894)	DUF2470, PyrE, EccD, ATPase, EsxA, EsxB, PPE, PE, EccC_b	312152……312442	312485…312775
*Mycobacterium branderi* JCM 12687 (AP022606)	No data available for the RD1 region	84207…84494	No ortholog
*Mycobacterium parmense* JCM 14742 (AP022614)	DNA binding, hypothetical protein, hypothetical protein, EccD1, ParA, EsxA, EsxB, PPE68, EccC_b	4342023…4342310	4342347…4342649
*Mycolicibacterium rhodesiae* NBB3 (CP003169)	EccC_b, PE family, PPE family, EsxB, EsxA, ATPase, EccD, DUF2580, hypothetical protein	1909036…1909320	1908694…1908999
*Mycobacteroides chelonae* NCTC 946 (LR134345)	EccC_b1, PE family, PPE family, EsxB, EsxA, ATPase, EccD1, Uncharacterised protein	50459…50746	50124…50426
*Mycolicibacterium phocaicum* JCM 15301(AP022616)	Peptidase, hypothetical protein, EccD, ParA, EsxA, EsxB, PPE family, PE35, EccC_b1	3692405…3692689	3692737…3693042
*Mycobacterium paragordonae* strain 49061 (CP025546)	EccC_b1, PPE_esxB, esxA, hypothetical protein, EccD, hypothetical protein, EspK	6685811…6686098	6685467…6685769
*Mycobacterium kubicae* strain JCM 13573 (CP065047)	EccC_b1, PPE family, EsxB, EsxA, ATPase, EccD, hypothetical protein, DUF1275 containing protein, EspK	5914823…5915110	5914484…5914786
*Mycolicibacterium frederiksbergense* strain LB 501T (CP038799)	Hypothetical protein, YbaB/Ebfc, EccD, ParA, EsxA, EsxB, PPE, PE, EccC_b1	2368442…2368727	2368768…2369061
*Mycobacterium vicinigordonae* strain 24 (CP059165)	EccC_b, PPE family, EsxB, EsxA, ATPase, EccD, hypothetical protein, EspK	6219197…6219484	6218855…6219157
*Mycobacterium shinjukuense* JCM 14233 (AP022575)	EccC_b, PPE68, EsxB, EsxA, hypothetical protein, hypothetical protein, EccD, hypothetical protein	3806327…3806614	3805993…3806295
*Mycobacterium seoulense* JCM 16018 (AP022582)	EccC_b, PPE68, EsxB, EsxA, hypothetical protein, EccD, hypotheical protein, esp protein	4173153…4173440	4172810…4173112
*Mycobacterium paraseoulense* JCM 16952 (AP022619)	EccC_b, PPE68, EsxB, EsxA, EspI, EccD, hypothetical protein, HTH, Esp protein	3978626…3978913	3978283…3978585
*Mycolicibacterium phlei* strain CCUG 21000 (CP014475)	EccC_b, PE35, PPE68, EsxB, EsxA, Ylxh, EccD, hypothetical protein, PknJ	50459…50746	50124…50426
*Mycolicibacterium flavescens* NCTC 10271 (LR134353)	EccC_b, uncharacterised protein, PE family, PPE family, EsxB, EsxA, ATPase, EccD, short chain dehydrogenase, acyl-CoA dehydrogenase	49302…49601	48974…49273
*Mycobacterium szulgai* NLA000501479 (FJ014490)	No genome data available	1–288	1–303
*Mycolicibacterium septicum* strain PDNC012 (CP070349)	EccC_b, PE family, PPE family, EsxB, EsxA, ParA, EccD, hypothetical protein, hypothetical protein	1169187…1169474	1168853…1169155
*Mycobacterium saskatchewanense* JCM 13016 (AP022573)	EccC_b, PPE family, EsxB, EsxA, EspI, EccD, hypothetical protein, hypothetical protein	3511060…3511347	3510717…3511019
*Mycolicibacterium mucogenicum* DSM 44124 (CP062008)	EccC_b, PE family, PPE family, EsxB, EsxA, ParA, EccD, hypothetical protein, metallopeptidase	69599…69883	69236…69541
*Mycobacterium gallinarum* JCM 6399 (AP022601)	EccC_b, PE35, PPE family, EsxB, EsxA, ParA, EccD, hypothetical protein WXG100 protein	4442297…4442593	4441952…4442266
*Mycobacterium kansasii* strain 9MK (CP019888)	EccC_b, PE family, PPE family, EsxB, EsxA, ATPase, EccD, hypothetical protein, EccK, EspK	3155767…3156054	3155428…3155730
*Mycobacterium lacus* JCM 15657 (AP022581)	Hypothetical protein, EspJ, EccD, hypothetical protein, esxA, EsxB, PPE68, PE35, EccC_b	4633999…4634286	4634323…4634623
*Mycobacterium riyadhense* NTM (CP045092)	No genome data available	162067…162354	161731…162031
*Mycolicibacterium* sp. TY81 (AP023362)	EccC_b, PE35, PPE family, EsxB, EsxA, ParA, EccD, hypothetical protein, LpqM	5159731…5160015	5159380…5159685
*Mycolicibacterium* sp. TY66 (AP023333)	LpqM, hypothetical protein, EccD, ParA, EsxA, EsxB, PPE family, PE35, EccC_b	341382…341666	341712…342017
*Mycobacterium marinum* M strain (CP000854)	EccC_b, PE family, PPE family, EsxB, EsxA, ATPase, EccD, hypothetical protein, hypothetical protein	6591497…6591784	6591158…6591460
*Mycobacterium shottsii* JCM 12657 (AP022572)	Hypothetical protein (espA_EspE), hypothetical protein, EccD, ATPase, EsxA, EsxB, PPE68, PE35, EccC_b	1084780…1085067	1085104…1085406
*Mycobacterium ulcerans* subsp *shinshuense* ATCC 33728 (AP017624)	(IS2404), PPE family, esxB, esxA, ATPase, transmebrane protein, hypothetical protein, (IS2404 (2) hypothetical protein	5856040…5856327	5855827…5856003
*Mycobacterium liflandii* 128FXT (CP003899)	EccC_b, (IS2404), PE family, PPE family, EsxB, EsxA, ATPase, EccD, Alanine rich protein, putative transmembrane protein	6154950…6155237	6154611…6154913
*Mycobacterium pseudoshotsii* JCM 15466 (AP018410)	EccC_b, PE35, PPE68, EsxB, EsxA, hypothetical protein, EccD, hypothetical protein, (ISAs1), hypothetical protein	6008914…6009201	6008575…6008877
*Mycolicibacterium gadium* JCM 12688 (AP022608)	Hypothetical protein, hypothetical protein, EccD, ParA, EsxA, EsxB, PPE family, PE35, EccC_b	4219252…4219548	4219579…4219890
*Mycobacterium leprae* TN (AL583917)	Hypothetical protein (Rv3879c pseudogene ortholog), hypothetical protein (pseudogen Rv3878 ortholog), membrane protein (Rv3877 ortholog), hypothetical protein (Rv3876 ortholog), EsxA, esxB, PPE family (Rv3873 ortholog), ATP binding protein (Rv 3871 ortholog)	61406…61693	61720…62022
*Mycolicibacterium gilvum* PYR-GCK (CP000656)	Hypothetical protein, hypothetical protein, ATPase, EsxA, EsxB, PPE family, PE family, FtsK/SpoIIIE	790048…790332	790366…790683
*Mycolicibacterium duvalii* JCM 6396 (AP022563)	YbaB/EbfC DNA-binding family, WXG100 protein, EccD, ParA, EsxA, EsxB, PPE family, PE35, EccC_b	4393935…4394222	4394254…4394556
*Mycolicibacterium poriferae* JCM 12603 (AP022570)	EccC_b, PE35, PPE family, EsxB, EsxA, ParA, EccD, hypothetical protein, hypothetical protein	664839…665092	664474…664779
*Mycolicibacterium aubagnense* JCM 15296 (AP022577)	EccC_b, PE35, PPE family, EsxB, EsxA, ParA, EccD, hypothetical protein, LpqM	3550625…3550915	3550272..3550577
*Mycolicibacterium malmesburyense* CIP:10822T (CVTB01000324)	EccC_b, hypothetical protein, PE family, PPE family, EsxB, EsxA, ATPase, EccD, DUF427, hypothetical protein	112653…112952	112325…112624
*Mycobacterium shimoidei* DSM 44152 (LQPU01000032.1)	Hypothetical protein, hypothetical protein, EccD, ParA, EsxA, EsxB, PPE family, PbsX family, EccC_b	128438…128725	128758……129060
*Mycolicibacterium brisbanense* JCM15654 (BCSX01000024)	LppB, EspA/EspB family, EccD, ATPase, EsxA, EsxB, PPE family, PE family, FtsK/SpoIIIE	595987…596274	596307…596606
*Mycobacterium haemophilum* DSM 44634 (CP011883)	EccC_b, PPE family, EsxB, EsxA, EspI, EccD, hypothetical protein, EspK	4192929…4193216	4192595…4192897
*Mycobacterium vanbaalenii* PYR-1 (CP000511)	FtsK/SpoIIIE, PE family, PPE family, EsxB, EsxA, ATPase, EccD, hypothetical protein, Esp protein	90169……90450	89821…90126
*Mycobacterium ostraviense* strain FDAARGOS_161.3 (CP089224)	EspK, EccD, ParA, hypothetical protein, EsxA, EsxB, PPE family, PE family, EccC_b	5591987…5592274	5592310…5592612
*Mycobacterium stomatepiae* JCM 17783 (AP022587)	Hypothetical protein, hypothetical protein, EccD, EspI, EsxA, EsxB, PPE family, EccC_b (possible pseudo)	4072234…4072527	4072571…4072882

## Data Availability

In this study, we used publicly available data which were accessed from NCBI (https://www.ncbi.nlm.nih.gov/), Smegmlist (https://mycobrowser.epfl.ch/genes/) and Bovilist (http://genolist.pasteur.fr/BoviList/genome.cgi?; accessed on 20 January 2022) databases. All other data generated or analysed in the current study are available upon request.

## Supplementary Materials

The following supporting information can be downloaded at: https://www.mdpi.com/article/10.3390/microorganisms12061151/s1, Figure S1: Gel electrophoresis image illustrating amplification of *esxA* gene fragment using *M. smegmatis* derived primers. Lane ‘M’ is 100 bp Molecular weight markers (O’ gene ruler from Thermofisher Scientific), Lanes 6, 7 and 8 are positive samples, Lanes 2, 3, 4, 9, 10,11,12,13,14, and 15 are negative samples, and lane PC is *M. smegmatis* positive control and lane NC1 and NC2 are negative controls. Figure S2: Gel electrophoresis image illustrating amplification of *esxB* gene fragment using *M. smegmatis* derived primers. Lane 1 is a positive sample, lane NC is a negative control, while lane PC is *M. smegmatis* positive control, and lane M is a 100 bp Molecular weight marker (100 bp O’gene Ruler from Thermo Fisher Scientific); Figure S3: Gel electrophoresis image illustrating amplification of *esxA* gene fragment using *M. bovis* derived primers. Lanes 1 and 4 are negative samples, Lane 2, is a positive sample and lane 3 is *M. bovis* control while lane 5 is a negative control, and lane M, a 100 bp Molecular weight marker (100 bp O’gene Ruler from Thermo Fisher Scientific); Figure S4: Gel electrophoresis image illustrating amplification of *esxB* gene fragment using *M. bovis* derived primers. Lanes 1 is *M. bovis* control, Lane 2, is a negative sample and lane 3 is a positive sample while lane 4 is a negative control, and lane M, a 100 bp Molecular weight marker (100 bp O’gene Ruler from Thermo Fisher Scientific).
