# Supplementary material for: The Presence of esat-6 and cfp10 and Other Gene Orthologs of the RD 1 Region in Non-Tuberculous Mycobacteria, Mycolicibacteria, Mycobacteroides and Mycolicibacter as Possible Impediments for the Diagnosis of (Animal) Tuberculosis
Source: Microorganisms. 2024 Jun 5;12(6):1151. doi: 10.3390/microorganisms12061151 (PMC11206017; doi:10.3390/microorganisms12061151)
Supplement: Supplementary file 1 [file microorganisms-12-01151-s001.zip › esxA fig S1.pptx]

## Slide 1
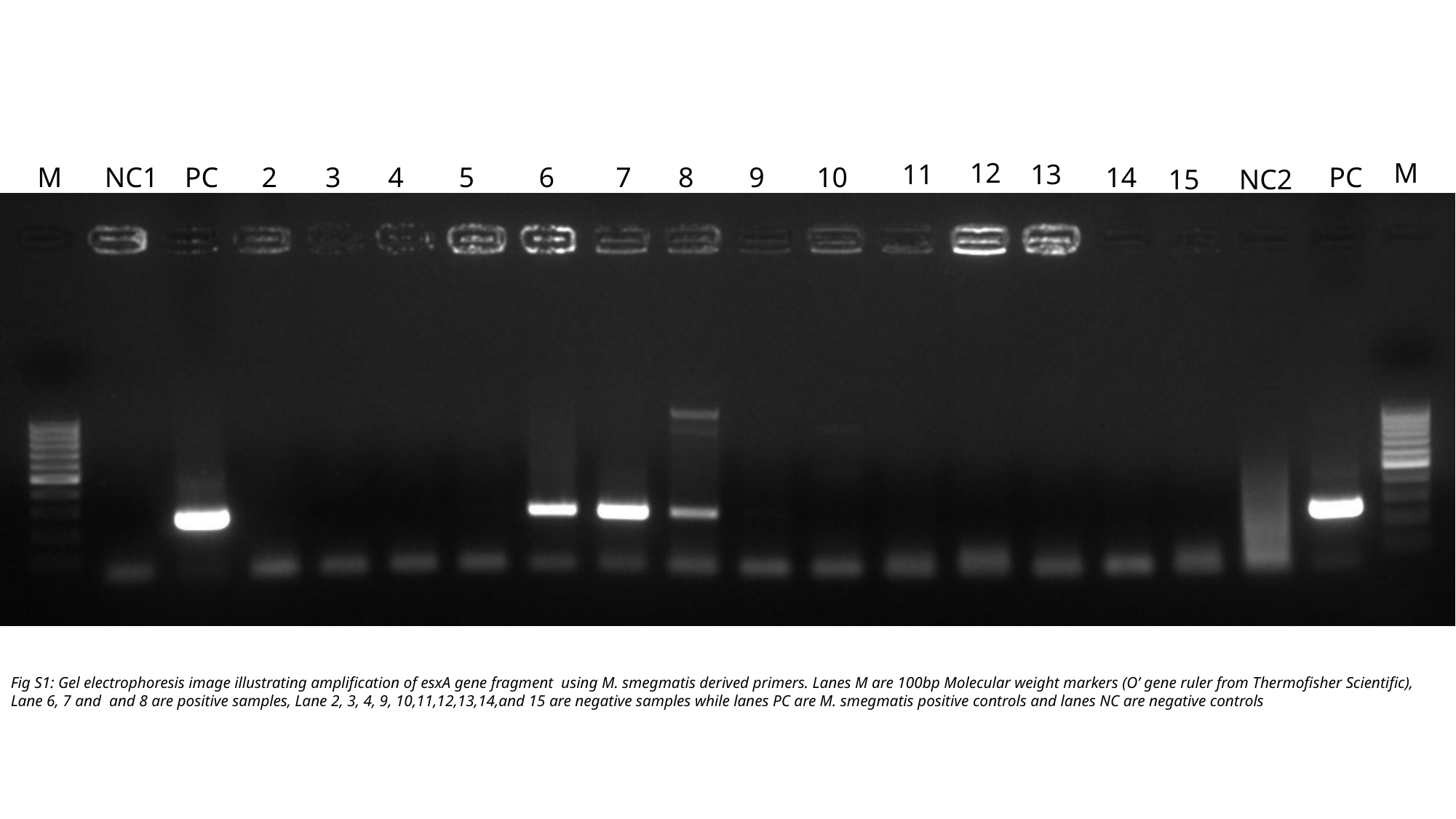

12
M
11
13
2
3
4
5
6
 7
8
9
10
PC
M
NC1
PC
14
15
NC2
Fig S1: Gel electrophoresis image illustrating amplification of esxA gene fragment using M. smegmatis derived primers. Lanes M are 100bp Molecular weight markers (O’ gene ruler from Thermofisher Scientific), Lane 6, 7 and and 8 are positive samples, Lane 2, 3, 4, 9, 10,11,12,13,14,and 15 are negative samples while lanes PC are M. smegmatis positive controls and lanes NC are negative controls
