# Supplementary material for: The Presence of esat-6 and cfp10 and Other Gene Orthologs of the RD 1 Region in Non-Tuberculous Mycobacteria, Mycolicibacteria, Mycobacteroides and Mycolicibacter as Possible Impediments for the Diagnosis of (Animal) Tuberculosis
Source: Microorganisms. 2024 Jun 5;12(6):1151. doi: 10.3390/microorganisms12061151 (PMC11206017; doi:10.3390/microorganisms12061151)
Supplement: Supplementary file 1 [file microorganisms-12-01151-s001.zip › Figure s 4.pptx]

## Slide 1
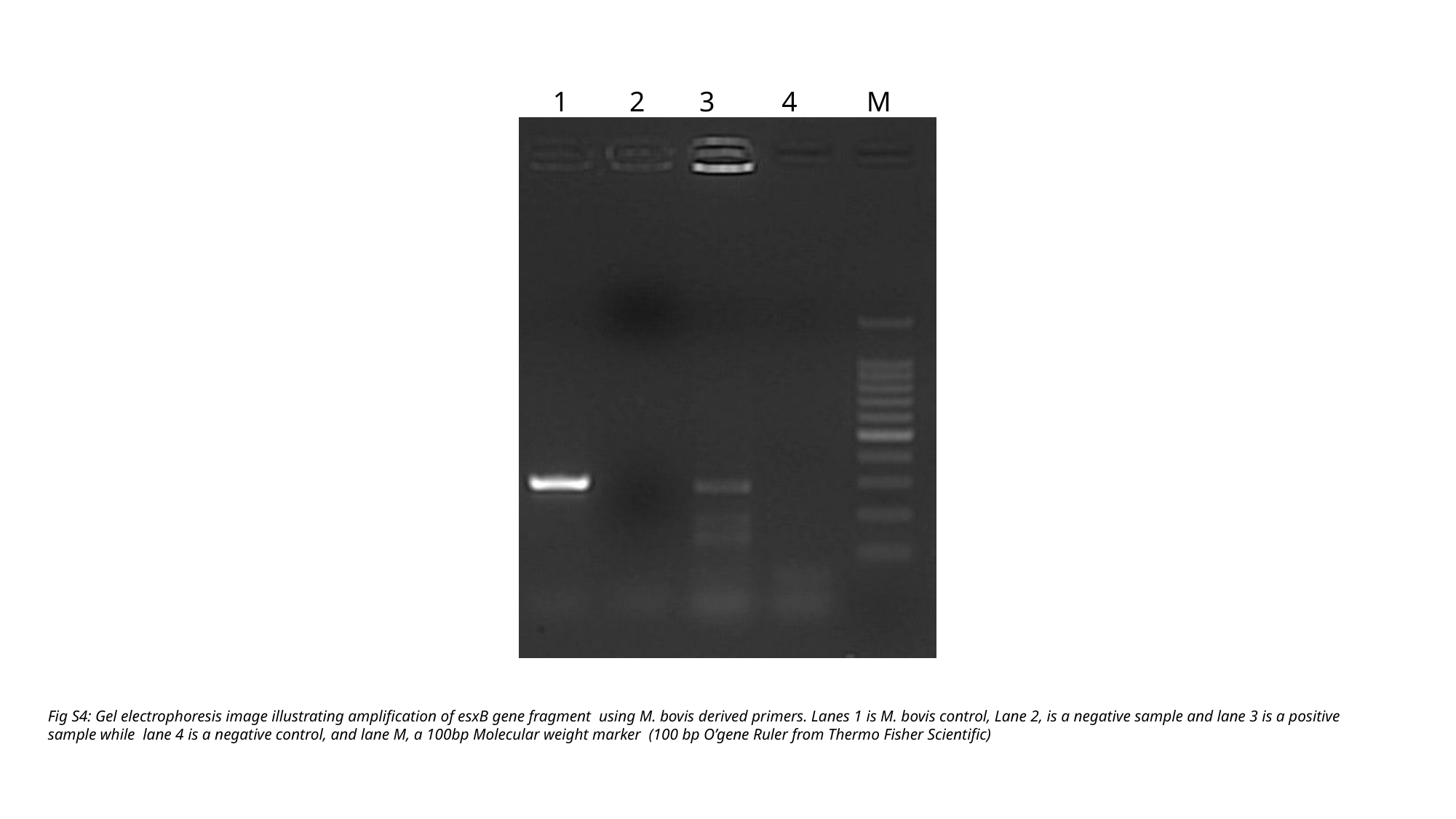

2
3
4
M
1
Fig S4: Gel electrophoresis image illustrating amplification of esxB gene fragment using M. bovis derived primers. Lanes 1 is M. bovis control, Lane 2, is a negative sample and lane 3 is a positive sample while lane 4 is a negative control, and lane M, a 100bp Molecular weight marker (100 bp O’gene Ruler from Thermo Fisher Scientific)
